# Electrophysiological changes between patients with suicidal ideation and suicide attempts: An event-related potential study

**DOI:** 10.1192/j.eurpsy.2023.1061

**Published:** 2023-07-19

**Authors:** S. Shim

**Affiliations:** Soonchunhyang University Cheonan Hospital, Cheonan, Korea, Republic Of

## Abstract

**Introduction:**

Suicide is recognized as a social problem and the interest in preventive measures to diminish suicide risk is constantly increasing. But scientific research results that distinguish between those who have only suicidal ideation (SI) and those who have a history of Suicidal attempts (SA) are limited. Inhibitory control is regarded as an important ability related to the transition from suicidal ideation to suicide attempts. In event-related potential, patients with dysfunction of inhibitory control demonstrate a reduction in the no-go amplitude.

**Objectives:**

This study aimed to determine the association between the no-go event-related potential component and suicidal behaviors among suicide attempters and ideators who never attempted suicide.

**Methods:**

Overall, 150 patients who visited the emergency room by suicide attempts or patients who visited the psychiatric department with suicidal ideation were recruited and instructed to perform a go/no-go task during electroencephalography recording. The Beck Depression Inventory, Beck Anxiety Inventory, Barratt Impulsivity Scale, Difficulties in Emotional Regulation Scale, and Acquired Capability for Suicide Scale were used. Individuals were divided into two groups: those with suicidal attempts (SA group) and with suicidal ideation (SI group) without SA. The psychological characteristics and event-related potentials of the two groups were compared. Correlation analyses were conducted to test the association between the clinical characteristics and event-related potentials.

**Results:**

The SA group had significantly decreased no-go P3 amplitudes at all electrodes compared to the SI group. In the correlation analysis between the clinical measurements and event-related potentials in all the participants, no-go P3 amplitudes in whole electrode sites were negatively correlated with the scores of the acquired capability for the suicide scale.

**Conclusions:**

This study revealed that suicide attempters have dysfunction in controlling inhibition compared to suicide ideators reflected in the no-go P3. Our findings suggested that no-go P3 can be a biomarker associated suicide attempts in suicide ideators.

**Disclosure of Interest:**

None Declared

